# The Chinese community patient’s life satisfaction, assessment of community medical service, and trust in community health delivery system

**DOI:** 10.1186/1477-7525-11-18

**Published:** 2013-02-13

**Authors:** Liyang Tang

**Affiliations:** 1 Department of Economics, School of Economics and Management, Tsinghua University, Beijing, 100084, China; 2 Graduate School of Business, Columbia University, New York, NY, 10027, USA

**Keywords:** Community health center, Community patient, Life satisfaction, Assessment of community medical service, Trust in community health delivery system, China

## Abstract

**Background:**

Although the Chinese government put a lot of effort into promoting the community patient’s life satisfaction, there still lacked the holistic and systematic approaches to promote the community patient’s life satisfaction in various regions of China. On the basis of the literature, it was found that both the community patient’s assessment of community medical service and trust in community health delivery system were important considerations when the community patient comprehensively evaluated community medical service to generate life satisfaction. So this study was set up to test whether and to what extent the community patient’s assessments of various major aspects of community medical service/various major aspects of the community patient’s trust in community health delivery system influenced life satisfaction in whole China/in various regions of China.

**Methods:**

In order to explore the situation of China’s community health delivery system before 2009 and provide a reference for China’s community health delivery system reform, the data that could comprehensively and accurately reflect the community patient’s life satisfaction, assessment of community medical service, and trust in community health delivery system in various regions of China was needed, so this study collaborated with the National Bureau of Statistics of China to carry out a large-scale 2008 national community resident household survey (N = 3,306) for the first time in China. And the specified ordered probit models were established to analyze the dataset from this household survey.

**Results:**

Among major aspects of community medical service, the medical cost (particularly in developed regions), the doctor-patient communication (particularly in developed regions), the medical facility and hospital environment (particularly in developed regions), and the medical treatment process (particularly in underdeveloped regions) were all key considerations (p<0.05 for t statistics) in generating the community patient’s life satisfaction. Among major aspects of the community patient’s trust in community health delivery system, trust in doctor (particularly in underdeveloped regions), trust in prescription (particularly in underdeveloped regions), and trust in recommended medical examination (particularly in underdeveloped regions) were all important considerations (p<0.10 for t statistics) in generating the community patient’s life satisfaction.

**Conclusion:**

The reduction of medical cost (particularly in developed regions), the improvement of doctor-patient communication (particularly in developed regions), the promotion of medical facility and hospital environment (particularly in developed regions), the improvement of medical treatment process (particularly in underdeveloped regions), the promotion of trust in doctor (particularly in underdeveloped regions), the promotion of trust in prescription (particularly in underdeveloped regions), and the promotion of trust in recommended medical examination (particularly in underdeveloped regions) could help promote the community patient’s life satisfaction.

## Background

The community health center (CHC) in China served as the complementary health care organization to the three-level public hospital system. Compared with hospitals in the three-level public hospital system, CHC mainly provided prevention service, treatment service, health promotion service, and rehabilitation service for participants of a defined community, and the featured CHC was composed of the clinical function area, the health promotion and epidemic prevention area, the medical support area, and the public area [1-3]. The establishment and development of CHC in China’s community health delivery system were speeded up after 2008, because the Chinese government put a lot of effort into promoting the community patient’s life satisfaction [4-6]. As the result, the participation of CHC in China’s health care market slightly alleviated the problem “medical service was expensive and difficult to access” [2], but the community patient was still not very satisfied with community medical service [4,7]. For example, in the 2008 national urban resident household survey, it was suggested that the quality of the process of delivering treatment service, the quality of doctor-patient communication, the quality of medical facility, and the quality of hospital environment in CHC should be further improved, and CHC should continue strengthening its competitive advantages (involving shorter waiting time for medical service and lower medical cost, compared with hospitals in the three-level public hospital system, here the definition of medical cost was the actual cost of providing both goods and services related to the delivery of medical care) to effectively promote the community patient’s life satisfaction [4,5].

Before 2009 the Chinese government set the policy objective that more than 50% of patients should potentially be triaged out from hospitals in the three-level public hospital system to go to CHC in order to ease overcrowding [8]. In order to achieve this policy objective, the Chinese government worked hard to increase the levels of both capital investment in CHC and training of community medical care staff [8]. However, this policy objective encountered the great challenge: only healthcare consumers that were poor, unemployed, or uninsured were inclined to choose CHC mostly because of economic concerns, while most patients in urban areas seldom went to CHC [4,8].

In order to solve the above challenge, the Chinese government started to implement the community health delivery system reform in 2009, and the aim of this reform was to transform the community medical service mode, continuously raise the service level, take the initiative to offer community medical service, provide household visit for community patient, and gradually assume the responsibility and duty of the “gate-keeper” for community patient’s health. But in the implementation of the community health delivery system reform, there still lacked the holistic and systematic approaches to promote the community patient’s life satisfaction in various regions of China [8].

On the basis of the literature, it was found that both the community patient’s assessment of community medical service and trust in community health delivery system were important considerations when the community patient comprehensively evaluated community medical service to generate life satisfaction. In fact it was widely accepted that the community patient generated multi-attributes based responses on life satisfaction [9-13]. One major part of the attribute-level responses of life satisfaction was composed of the community patient’s assessments of various major aspects of community medical service, and the reason for this was that the community patient’s life satisfaction could be seen as a comprehensive response that resulted from both pre-treatment expectation and post-treatment cognitive and affective evaluation of community medical service [14-22]. On the basis of previous studies, the major aspects of community medical service consisted of the quality of medical treatment process, the quality of doctor-patient communication, the length of waiting time for medical service, the quantity and quality of medical facility, the quality of hospital environment, and the medical cost [4,5,23-27]. Another major part of the attribute-level responses of life satisfaction was composed of various major aspects of the community patient’s trust in community health delivery system, because when the community patient formed the stable trust in community health delivery system as a response to the unbalanced relationship between community patient and community doctor/CHC, the community patient’s trust in community health delivery system influenced attitude towards, cognition on, and response mode with the effectiveness of community medical service to a large extent [28-34]. In literature, the most important aspects of the community patient’s trust in community health delivery system consisted of trust in medical institution, trust in doctor, trust in prescription, and trust in recommended medical examination [4,5,23,25,35]. Other parts of the attribute-level responses of life satisfaction were composed of the community patient’s certain personal characteristics, for example, the community patient’s demographic characteristics, socioeconomic characteristics, medical insurance status, health status, and disease status were all proved to be the possible influencing factors for life satisfaction [4,5].

The reasoning behind the study design was as follows. As many researchers pointed out, there lacked the holistic and systematic approaches to promote the community patient’s life satisfaction in various regions of China [4-6,8,26,27,35]. On the basis of the literature, it was found that both the promotion of key aspects of community medical service and the promotion of key aspects of the community patient’s trust in community health delivery system were usually the indirect but effective approaches to promote the community patient’s life satisfaction in China’s community health delivery system [4,5]. But there was a big problem with these effective approaches in China: developed regions usually had more abundant community medical resources than underdeveloped regions, and the establishment and development of CHC in different regions of China were also significantly different, so the effective approaches to promote the community patient’s life satisfaction might vary across regions. And then this study not only needed to explore the effective approaches to promote the community patient’s life satisfaction in whole China, but also needed to explore various effective approaches to promote the community patient’s life satisfaction in various regions of China.

In order to test whether and to what extent the community patient’s assessments of various major aspects of community medical service/various major aspects of the community patient’s trust in community health delivery system influenced life satisfaction in whole China/in various regions of China, this study collaborated with the National Bureau of Statistics of China to carry out a large-scale 2008 national community resident household survey for the first time in China, and the specified ordered probit models were established to analyze the dataset from this household survey. On the basis of the major findings of this study, the inspirations on the effective approaches to promote the community patient’s life satisfaction in whole China/in various regions of China were also found for China’s future community health delivery system reform.

## Methods

### Data

In order to explore the situation of China’s community health delivery system before 2009 and provide a reference for China’s community health delivery system reform, the data that could comprehensively and accurately reflect the community patient’s life satisfaction, assessment of community medical service, and trust in community health delivery system in various regions of China was needed, so this study collaborated with the National Bureau of Statistics of China to carry out a large-scale 2008 national community resident household survey in 18 provinces, autonomous regions, and municipalities directly under the central government.

In this household survey, the National Bureau of Statistics of China adopted the most strict two-stage probability proportional to size (PPS) systematic sampling technique to select a probability sample of 3,600 community residents, and the advantage of this technique was that it could select a perfect stratified sample, but its disadvantage was that any deviation from the stratified sampling design could impact the effect of stratification. The face-to-face interviews for this household survey were conducted by the professional survey teams from the National Bureau of Statistics, local Bureaus of Statistics, and Tsinghua University. The professional investigator usually first invited the selected community resident to fill out the questionnaire of this household survey, no replacement was made if the selected community resident was away, refused to be interviewed, or failed to be interviewed after three attempts. If the selected community resident agreed to fill out the questionnaire of this household survey, but she/he was unavailable, or disabled in a way that impeded her/him from filling out the questionnaire, another family member that knew her/him best served as the respondent, and this family member was also asked to report her/his assessed values of questions in the questionnaire to check bias. A total of 3,306 valid responses were generated in the 2008 national community resident household survey, and the response rate was 91.83%.

The questionnaire of this household survey consisted of many sections, among them four sections had relation to this study. The first section inquired about the community resident’s certain personal characteristics (involving age, gender, marital status, education, income, employment status, occupation, health status, medical insurance, reimbursement percentage of medical cost, severity of disease, and stage of disease in the most recent community medical experience), and all these selected personal characteristics were considered as the possible influencing factors for the community resident’s life satisfaction in the most recent community medical experience. The second section inquired about the community resident’s life satisfaction in the most recent community medical experience. The third section inquired about the community resident’s assessment of community medical service in the most recent community medical experience, and the selection of five major aspects of community medical service was based on the top five aspects of community medical service that the community resident was most concerned about in the pre-survey for this study [4,5], specifically speaking, the community resident’s assessments of five major aspects of community medical service consisted of assessment of medical treatment process, assessment of doctor-patient communication, assessment of waiting time for medical service, assessment of medical facility and hospital environment, and assessment of medical cost. The fourth section inquired about the community resident’s trust in community health delivery system in the most recent community medical experience, the selection of four major aspects of the community resident’s trust in community health delivery system was based on the interview materials in the pre-survey for this study [4,5], and four major aspects of the community resident’s trust in community health delivery system were composed of trust in medical institution, trust in doctor, trust in prescription, and trust in recommended medical examination.

The personal characteristics of the study population were shown in Table 1. As the result of the stratified sampling design by the National Bureau of Statistics of China, the population distribution of each personal characteristic followed the natural distribution of community resident in China. The data in this household survey was collected anonymously, and the use of the dataset in this study was approved by the National Bureau of Statistics of China.

**Table 1:** Descriptive statistics of personal characteristics

**Dummy variables**	**Descriptions**	**Mean**	**Standard deviation**	**Min**	**Max**
Age dummy variables	Sample person is between 18–30 years old, 1=Yes, 0=Otherwise	0.276	0.447	0	1
	Sample person is between 31–45 years old, 1=Yes, 0=Otherwise	0.247	0.431	0	1
	Sample person is between 46–55 years old, 1=Yes, 0=Otherwise	0.244	0.430	0	1
Gender dummy variable	1=Male, 0=Female	0.485	0.500	0	1
Marital status dummy variables	1=Unmarried, 0=Otherwise	0.015	0.120	0	1
	1=Married, 0=Otherwise	0.889	0.315	0	1
	1=Divorced, 0=Otherwise	0.037	0.189	0	1
	1=Widowed, 0=Otherwise	0.056	0.231	0	1
Education dummy variables	1=Primary school or below, 0=Otherwise	0.098	0.298	0	1
	1=Junior high school, 0=Otherwise	0.285	0.452	0	1
	1=Senior high school, 0=Otherwise	0.268	0.443	0	1
	1=Secondary, 0=Otherwise	0.111	0.315	0	1
	1=College, 0=Otherwise	0.148	0.356	0	1
	1=University, 0=Otherwise	0.082	0.275	0	1
Income dummy variables	1=Income is less than ¥3390, 0=Otherwise	0.111	0.315	0	1
	1=Income is between ¥3390 and ¥5410, 0=Otherwise	0.102	0.302	0	1
	1=Income is between ¥5411 and ¥7420, 0=Otherwise	0.134	0.341	0	1
	1=Income is between ¥7421 and ¥9374, 0=Otherwise	0.148	0.356	0	1
	1=Income is between ¥9375 and ¥11700, 0=Otherwise	0.119	0.324	0	1
	1=Income is between ¥11701 and ¥15180, 0=Otherwise	0.148	0.356	0	1
	1=Income is between ¥15181 and ¥21860, 0=Otherwise	0.118	0.323	0	1
Employment status dummy variables	1=Employees of state-owned enterprises, 0=Otherwise	0.289	0.454	0	1
	1=Employees of various non-state-owned enterprises, 0=Otherwise	0.216	0.412	0	1
	1=Urban self-employed and private entrepreneurs, 0=Otherwise	0.063	0.243	0	1
	1=Homeworkers, 0=Otherwise	0.253	0.435	0	1
	1=Unemployed, to be distributed or other non-employed, 0=Otherwise	0.016	0.126	0	1
	1=Students, 0=Otherwise	0.045	0.208	0	1
	1=Reemployment of retired or retired personnel, 0=Otherwise	0.035	0.185	0	1
Occupation dummy variables	1=Professional and technical personnel, 0=Otherwise	0.018	0.132	0	1
	1=Managers in government and government related enterprises, 0=Otherwise	0.127	0.334	0	1
	1=The clerk and manager, 0=Otherwise	0.208	0.406	0	1
	1=Commercial staff, 0=Otherwise	0.139	0.346	0	1
	1=Service staff, 0=Otherwise	0.008	0.090	0	1
	1=Farmers, animal husbandry and fishery workers, 0=Otherwise	0.110	0.313	0	1
	1=Production workers, transport workers and associated personnel, 0=Otherwise	0.002	0.040	0	1
Health status dummy variable	1=Health status is at average level or above average level, 0=Otherwise	0.926	0.262	0	1
Medical insurance dummy variables	1=Medical insurance for local urban workers, 0=Otherwise	0.535	0.499	0	1
	1=Medical insurance for local migrant workers, 0=Otherwise	0.003	0.057	0	1
	1=Self-financing medical insurance sponsored by the company or unit, 0=Otherwise	0.047	0.211	0	1
	1=Commercial medical insurance bought by employer, 0=Otherwise	0.006	0.080	0	1
	1=Privately purchased commercial medical insurance, 0=Otherwise	0.056	0.231	0	1
	1=Government funded health care reimbursement, 0=Otherwise	0.042	0.201	0	1
	1=The new rural cooperative medical insurance, 0=Otherwise	0.065	0.246	0	1
	1=Other medical insurance, 0=Otherwise	0.095	0.294	0	1
	1=No medical insurance, 0=Otherwise	0.140	0.348	0	1
Reimbursement percentage of medical cost dummy variables	1=Reimbursement percentage of medical cost is 100%, 0=Otherwise	0.089	0.285	0	1
	1=Reimbursement percentage of medical cost is between 70% and 99%, 0=Otherwise	0.170	0.376	0	1
	1=Reimbursement percentage of medical cost is between 40% and 69%, 0=Otherwise	0.104	0.305	0	1
	1=Reimbursement percentage of medical cost is between 20% and 39%, 0=Otherwise	0.052	0.222	0	1
	1=Reimbursement percentage of medical cost is between 1% and 19%, 0=Otherwise	0.040	0.197	0	1
Severity of disease dummy variables	1=Not serious, 0=Otherwise	0.371	0.483	0	1
	1=General, 0=Otherwise	0.555	0.497	0	1
	1=Serious, 0=Otherwise	0.055	0.228	0	1
Stage of disease dummy variables	1=Emergency and serious disease, 0=Otherwise	0.079	0.270	0	1
	1=Non-emergency disease at initial stage, 0=Otherwise	0.706	0.456	0	1
	1=Non-emergency disease at medium stage, 0=Otherwise	0.106	0.309	0	1
	1=Non-emergency stable disease at late stage, 0=Otherwise	0.108	0.311	0	1

### Classification of regions

According to the regional per capita GDP and the richness of regional community medical resources in 2008, 18 provinces, autonomous regions, and municipalities directly under the central government in the 2008 national community resident household survey were divided into 3 groups by the National Bureau of Statistics of China:

1. Regions with the highest per capita GDP and the most abundant community medical resources (Group 1): Beijing, Shanghai;

2. Regions with the second highest per capita GDP and the second most abundant community medical resources (Group 2): Guangdong, Tianjin, Zhejiang, Fujian;

3. Regions with the lowest per capita GDP and the least abundant community medical resources (Group 3): Hubei, Hunan, Anhui, Heilongjiang, Jilin, Chongqing, Guangxi, Guizhou, Shaanxi, Ningxia, Qinghai, Xizang.

### Measure of the community patient’s life satisfaction

The 5-item self-reporting measure that assessed the community patient’s life satisfaction in the most recent community medical experience on a scale of 1 to 5 was employed, and the higher score reflected the community patient’s higher level of life satisfaction. In this study, the definition of satisfaction was a measure of how medical goods and medical services met or surpassed the community patient’s expectation, and then life satisfaction was operationalized as satisfaction with health-related quality of life and measured with the following question “If you compared your life after the most recent community medical experience with your life before the most recent community medical experience, was your health-related quality of life after the most recent community medical experience better than, equal to, or worse than that before the most recent community medical experience?”. The option “Much better” was assigned score 5; the option “Somewhat better” was assigned score 4; and the option “Equal” was assigned score 3; the option “Somewhat worse” was assigned score 2; and the option “Much worse” was assigned score 1.

### Measure of the community patient’s assessment of community medical service

The 5-item self-reporting measures that assessed the medical treatment process, the doctor-patient communication, the waiting time for medical service, the medical facility and hospital environment, and the medical cost in the community patient’s most recent community medical experience on a scale of 1 to 5 were employed, and the higher score reflected the community patient’s higher rating for a certain aspect of community medical service: the option “Excellent performance in this aspect” was assigned score 5; the option “Good performance in this aspect” was assigned score 4; and the option “General performance in this aspect” was assigned score 3; the option “Bad performance in this aspect” was assigned score 2; and the option “Poor performance in this aspect” was assigned score 1. Here the definition of performance in this study was the extent of the execution or accomplishment of community medical service related work.

### Measure of the community patient’s trust in community health delivery system

The 5-item self-reporting measures that assessed the community patient’s trust in medical institution, trust in doctor, trust in prescription, and trust in recommended medical examination in the most recent community medical experience on a scale of 1 to 5 were employed, in this study the definition of trust was the trait of believing in the honesty and reliability of the target object, and the higher score reflected the community patient’s higher degree of trust in a certain aspect of community health delivery system: the option “The high degree of trust in this aspect” was assigned score 5; the option “The relatively high degree of trust in this aspect” was assigned score 4; and the option “The medium degree of trust in this aspect” was assigned score 3; the option “The relatively low degree of trust in this aspect” was assigned score 2; and the option “The low degree of trust in this aspect” was assigned score 1.

### Description of ordered probit model

Due to the fact that the ordered probit was the sole method that discerned unequal differences between ordinal categories in the dependent variable-the community patient’s life satisfaction, the ordered probit model was the sole solution to the research question of this study [36-38]. For example, the ordered probit model didn’t assume that the difference between choosing “Much better” and choosing “Somewhat better” was the same as the difference between choosing “Somewhat worse” and choosing “Much worse”. In fact the ordered probit in this study captured the qualitative differences between different levels of life satisfaction.

In the ordered probit model, the latent evaluation score y_i_ was a linear function of independent variables that were written as a vector x_i_, here i was the sample number, and y_i_=x_i_*b+ε_i_, b was a vector of coefficients, and ε_i_ was assumed to follow a standard normal distribution. When an ordered probit model with k cutoff points was established, p_j_ (j=1,2,…,k) were defined as the cutoff points of all y_i_, then y_i_≦p_1_, p_j_<y_i_≦p_j+1_ (j=1,2,…,k-1) or y_i_>p_k_. Following the notation, the ordered probit model was expressed as

(1) $$\mathrm{Prob}\left(y_{i}=y_{0}|x_{i}\right)=Ф\left(p_{1}-x_{i}*b\right)$$

(2) $$\begin{array}{l} \mathrm{Prob}\left(y_{i}=y_{j}|x_{i}\right)=Ф\left(p_{j+1}-x_{i}*b\right)-Ф\left(p_{j}-x_{i}*b\right)\\ \;\left(j=1,2,\ldots ,k-1\right) \end{array}$$

(3) $$\mathrm{Prob}\left(y_{i}=y_{k}|x_{i}\right)=1-Ф\left(p_{k}-x_{i}*b\right)$$

here y_j_ (j=0,1,…k) were the discrete values of y_i_ and Ф was the standard normal cumulative distribution function [3,39].

The marginal effect of x_i_ could be calculated according to this formula:

(4) $$\partial \mathrm{Prob}\left(y_{i}=y_{0}|x_{i}\right)/\partial x_{i}=-b*\varphi \left(p_{1}-x_{i}*b\right)$$

(5) $$\begin{array}{l} \partial \mathrm{Prob}\left(y_{i}=y_{j}|x_{i}\right)/\partial x_{i}=-b*\left[\varphi \left(p_{j+1}-x_{i}*b\right)\right)\\ \;\left(-\varphi \left(p_{j}-x_{i}*b\right)\right]\\ \;\left(j=1,2,\ldots ,k-1\right) \end{array}$$

(6) $$\partial \mathrm{Prob}\left(y_{i}=y_{k}|x_{i}\right)/\partial x_{i}=b*\varphi \left(p_{k}-x_{i}*b\right)$$

here was the standard normal density function, and based on (4), (5) and (6) the vector of coefficients b could be estimated [3,39].

### Specified ordered probit models

The first specified ordered probit model was estimated with respect to all samples/the samples in various groups in order to test whether and to what extent the community patient’s assessments of various major aspects of community medical service influenced life satisfaction in whole China/in various regions of China:

(7) $$\begin{array}{l} \mathrm{Model}\;1:{\mathrm{satisfaction}}_{i}\\ \;=\sum_{l}\beta_{l1}{\mathrm{assessment}}_{\mathrm{li}}+\sum_{m}\beta_{m2}z_{\mathrm{mi}}+\varepsilon_{i} \end{array}$$

here i was the sample number; satisfaction_i_ was the community patient’s life satisfaction; assessment_li_ (l=1,2,…,5) were the community patient’s assessment of medical treatment process, assessment of doctor-patient communication, assessment of waiting time for medical service, assessment of medical facility and hospital environment, and assessment of medical cost; z_mi_ were control variables, since the community patient’s life satisfaction in the most recent community medical experience might be influenced by certain personal characteristics (involving age, gender, marital status, education, income, employment status, occupation, health status, medical insurance, reimbursement percentage of medical cost, severity of disease, and stage of disease in the most recent community medical experience), all selected personal characteristics were controlled as dummy variables in the regression model in order to address the interaction between social demographic figures and geographic variations; error term ε_i_ was assumed to follow a standard normal distribution.

The second specified ordered probit model was estimated with respect to all samples/the samples in various groups in order to test whether and to what extent various major aspects of the community patient’s trust in community health delivery system influenced life satisfaction in whole China/in various regions of China:

(8) $$\begin{array}{l} \mathrm{Model}\;2:{\mathrm{satisfaction}}_{i}\\ \;=\sum_{n}\beta_{n1}{\mathrm{trust}}_{\mathrm{ni}}+\sum_{m}\beta_{m2}z_{\mathrm{mi}}+\varepsilon_{i} \end{array}$$

here trust_ni_ (n=1,2,…,4) were the community patient’s trust in medical institution, trust in doctor, trust in prescription, and trust in recommended medical examination; the descriptions of other variables were the same as those in the first specified ordered probit model.

## Results

### Descriptive statistics

The descriptive statistics of the community patient’s life satisfaction, assessment of community medical service, and trust in community health delivery system were presented in Table 2. Generally speaking, the mean value of the community patient’s life satisfaction was between the assigned value for the option “Health-related quality of life after the most recent community medical experience was somewhat better than that before the most recent community medical experience” and the assigned value for the option “Health-related quality of life after the most recent community medical experience was equal to that before the most recent community medical experience”. Among the community patient’s assessments of various major aspects of community medical service, the mean value of the community patient’s assessment of medical treatment process was the highest, the mean values of the community patient’s assessment of waiting time for medical service and assessment of doctor-patient communication were the second highest, and the mean value of the community patient’s assessment of medical cost was in the medium level, while the mean value of the community patient’s assessment of medical facility and hospital environment was the lowest. The standard deviation of the community patient’s assessment of waiting time for medical service was significantly higher than the standard deviations of the community patient’s assessments of other aspects of community medical service. Among various major aspects of the community patient’s trust in community health delivery system, the mean values of the community patient’s trust in recommended medical examination and trust in doctor were the highest, and the mean value of the community patient’s trust in prescription was in the medium level, while the mean value of the community patient’s trust in medical institution was the lowest. The standard deviations of the community patient’s trust in doctor and trust in medical institution were significantly higher than the standard deviations of the community patient’s trust in prescription and trust in recommended medical examination.

**Table 2:** Descriptive statistics of the community patient’s life satisfaction, assessment of community medical service, and trust in community health delivery system

	**Mean**	**Standard deviation**	**Min**	**Max**
Life satisfaction	3.765	0.684	1	5
Assessment of medical treatment process	3.695	0.740	1	5
Assessment of doctor-patient communication	3.550	0.754	1	5
Assessment of waiting time for medical service	3.576	1.023	1	5
Assessment of medical facility and hospital environment	3.273	0.616	1	5
Assessment of medical cost	3.466	0.740	1	5
Trust in medical institution	2.321	0.945	1	5
Trust in doctor	4.121	0.983	1	5
Trust in prescription	3.481	0.883	1	5
Trust in recommended medical examination	4.368	0.851	1	5

### Regression results with respect to all samples

The results of both model 1 and model 2 with respect to all samples were presented in Table 3. Among the community patient’s assessments of various major aspects of community medical service, assessment of medical cost and assessment of doctor-patient communication had the largest positive influences on the community patient’s life satisfaction, assessment of medical facility and hospital environment and assessment of medical treatment process had the second largest positive influences on the community patient’s life satisfaction, while assessment of waiting time for medical service had no significant influence on the community patient’s life satisfaction. Among various major aspects of the community patient’s trust in community health delivery system, trust in doctor had the largest positive influence on the community patient’s life satisfaction, trust in prescription had the second largest positive influence on the community patient’s life satisfaction, and trust in recommended medical examination had the third largest positive influence on the community patient’s life satisfaction, while trust in medical institution had no significant influence on the community patient’s life satisfaction.

**Table 3:** Results of both model 1 and model 2 with respect to all samples

	**Model 1**	**Model 2**
	**Life satisfaction**	
Assessment of medical treatment process	0.264**	
	(2.21)	
Assessment of doctor-patient communication	0.310**	
	(2.53)	
Assessment of waiting time for medical service	−0.00362	
	(−0.05)	
Assessment of medical facility and hospital environment	0.273**	
	(2.27)	
Assessment of medical cost	0.319***	
	(2.89)	
Trust in medical institution		0.0400
		(0.77)
Trust in doctor		0.195***
		(3.94)
Trust in prescription		0.130**
		(2.37)
Trust in recommended medical examination		0.107*
		(1.88)
Age dummy variables	Yes	Yes
Gender dummy variable	Yes	Yes
Marital status dummy variables	Yes	Yes
Education dummy variables	Yes	Yes
Income dummy variables	Yes	Yes
Employment status dummy variables	Yes	Yes
Occupation dummy variables	Yes	Yes
Health status dummy variable	Yes	Yes
Medical insurance dummy variables	Yes	Yes
Reimbursement percentage of medical cost dummy variables	Yes	Yes
Severity of disease dummy variables	Yes	Yes
Stage of disease dummy variables	Yes	Yes
Cutoff point 1	0.908	−0.296
	(0.55)	(−0.20)
Cutoff point 2	2.287	1.337
	(1.39)	(0.93)
Cutoff point 3	3.115*	2.100
	(1.90)	(1.46)
Cutoff point 4	5.859***	4.515***
	(3.51)	(3.13)
Number of observations	3306	3306
Log pseudo-likelihood	102.7	67.14

t statistics in parentheses, * p < 0.10, ** p < 0.05, *** p < 0.01.

### Regression results with respect to the samples in various groups

The results of model 1 with respect to the samples in various groups were presented in Table 4. The community patient’s assessment of medical cost, assessment of doctor-patient communication, assessment of medical facility and hospital environment, and assessment of medical treatment process had significant positive influences on life satisfaction in all groups, but the community patient’s assessment of waiting time for medical service had no significant influence on life satisfaction in any group. The positive influences of the community patient’s assessment of medical cost, assessment of doctor-patient communication, and assessment of medical facility and hospital environment on life satisfaction in regions with higher per capita GDP and more abundant community medical resources were larger than those in regions with lower per capita GDP and less abundant community medical resources. The positive influence of the community patient’s assessment of medical treatment process on life satisfaction in regions with lower per capita GDP and less abundant community medical resources was larger than that in regions with higher per capita GDP and more abundant community medical resources.

**Table 4:** Results of model 1 with respect to the samples in various groups

	**(1)**	**(2)**	**(3)**
	**Life satisfaction**		
	**Group 1**	**Group 2**	**Group 3**
Assessment of medical treatment process	0.100*	0.510**	0.681***
	(1.70)	(2.06)	(2.74)
Assessment of doctor-patient communication	0.652***	0.325***	0.190*
	(3.48)	(2.76)	(1.84)
Assessment of waiting time for medical service	0.103	−0.191	−0.198
	(1.02)	(−1.31)	(−0.74)
Assessment of medical facility and hospital environment	0.382**	0.231**	0.148*
	(2.37)	(2.05)	(1.86)
Assessment of medical cost	0.513***	0.387**	0.286**
	(3.44)	(2.39)	(2.24)
Age dummy variables	Yes	Yes	Yes
Gender dummy variable	Yes	Yes	Yes
Marital status dummy variables	Yes	Yes	Yes
Education dummy variables	Yes	Yes	Yes
Income dummy variables	Yes	Yes	Yes
Employment status dummy variables	Yes	Yes	Yes
Occupation dummy variables	Yes	Yes	Yes
Health status dummy variable	Yes	Yes	Yes
Medical insurance dummy variables	Yes	Yes	Yes
Reimbursement percentage of medical cost dummy variables	Yes	Yes	Yes
Severity of disease dummy variables	Yes	Yes	Yes
Stage of disease dummy variables	Yes	Yes	Yes
Cutoff point 1	2.631**	2.073	2.164
	(2.05)	(1.18)	(0.69)
Cutoff point 2	4.006**	3.365*	3.656
	(2.08)	(1.93)	(1.15)
Cutoff point 3	4.870**	4.132**	4.742**
	(2.53)	(2.36)	(2.18)
Cutoff point 4	7.897***	6.691***	8.199**
	(3.98)	(3.68)	(2.21)
Number of observations	1106	916	1284
Log pseudo-likelihood	77.76	29.62	31.21

t statistics in parentheses, * p < 0.10, ** p < 0.05, *** p < 0.01.

The results of model 2 with respect to the samples in various groups were presented in Table 5. The community patient’s trust in doctor, trust in prescription, and trust in recommended medical examination had significant positive influences on life satisfaction in all groups, but the community patient’s trust in medical institution had no significant influence on life satisfaction in any group. The positive influences of the community patient’s trust in doctor, trust in prescription, and trust in recommended medical examination on life satisfaction in regions with lower per capita GDP and less abundant community medical resources were larger than those in regions with higher per capita GDP and more abundant community medical resources.

**Table 5:** Results of model 2 with respect to the samples in various groups

	**(1)**	**(2)**	**(3)**
	**Life satisfaction**		
	**Group 1**	**Group 2**	**Group 3**
Trust in medical institution	0.0348	0.101	−0.0694
	(0.49)	(1.05)	(−0.47)
Trust in doctor	0.170*	0.184***	0.407***
	(1.76)	(2.76)	(2.92)
Trust in prescription	0.0973*	0.119**	0.268**
	(1.81)	(1.98)	(2.13)
Trust in recommended medical examination	0.0951*	0.123**	0.248***
	(1.82)	(2.25)	(2.61)
Age dummy variables	Yes	Yes	Yes
Gender dummy variable	Yes	Yes	Yes
Marital status dummy variables	Yes	Yes	Yes
Education dummy variables	Yes	Yes	Yes
Income dummy variables	Yes	Yes	Yes
Employment status dummy variables	Yes	Yes	Yes
Occupation dummy variables	Yes	Yes	Yes
Health status dummy variable	Yes	Yes	Yes
Medical insurance dummy variables	Yes	Yes	Yes
Reimbursement percentage of medical cost dummy variables	Yes	Yes	Yes
Severity of disease dummy variables	Yes	Yes	Yes
Stage of disease dummy variables	Yes	Yes	Yes
Cutoff point 1	1.311	0.954	1.579
	(0.84)	(0.62)	(0.94)
Cutoff point 2	2.055	2.427	2.656
	(1.31)	(1.61)	(1.57)
Cutoff point 3	4.536***	3.191**	4.129***
	(2.87)	(2.11)	(3.01)
Cutoff point 4	5.338***	5.498***	5.490***
	(3.62)	(3.56)	(3.12)
Number of observations	1106	916	1284
Log pseudo-likelihood	30.53	25.36	23.12

t statistics in parentheses, * p < 0.10, ** p < 0.05, *** p < 0.01.

## Discussion

### Major findings of this study

In China’s community health delivery system, among five major aspects of community medical service, the medical cost (particularly in regions with higher per capita GDP and more abundant community medical resources), the doctor-patient communication (particularly in regions with higher per capita GDP and more abundant community medical resources), the medical facility and hospital environment (particularly in regions with higher per capita GDP and more abundant community medical resources), and the medical treatment process (particularly in regions with lower per capita GDP and less abundant community medical resources) were all key considerations in generating the community patient’s life satisfaction. Among four major aspects of the community patient’s trust in community health delivery system, trust in doctor (particularly in regions with lower per capita GDP and less abundant community medical resources), trust in prescription (particularly in regions with lower per capita GDP and less abundant community medical resources), and trust in recommended medical examination (particularly in regions with lower per capita GDP and less abundant community medical resources) were all important considerations in generating the community patient’s life satisfaction.

### What is already known on this topic

The previous studies separately showed that the community patient’s assessment of community medical service and trust in community health delivery system were important considerations when the community patient comprehensively evaluated community medical service to generate life satisfaction [5,6,23-27,35]. But the community patient’s assessment of community medical service/the community patient’s trust in community health delivery system was usually taken as a whole in most of these previous studies, while few studies subdivided the community patient’s assessment of community medical service/the community patient’s trust in community health delivery system into various aspects and further explored the differences of their influencing effects on the community patient’s life satisfaction [5,6,23-27,35]. And there were few studies that explored the regional differences of the influences of both the community patient’s assessment of community medical service and trust in community health delivery system on life satisfaction in China.

Most previous studies obtained their conclusions only through the qualitative analysis or the authors’ experiences, while only a small number of previous studies adopted the strict quantitative analysis, but their conclusions were usually drawn on the basis of a small range of community patients, then the robustness and universality of their conclusions remained controversial.

### What this study adds

The most important contribution of this study was that the influences of the community patient’s assessments of various major aspects of community medical service/various major aspects of the community patient’s trust in community health delivery system on life satisfaction in whole China/in various regions of China before 2009 were studied under a systematic and comprehensive framework for the first time. The second most important contribution of this study was that in order to perform the strict quantitative analysis, this study collaborated with the National Bureau of Statistics of China to carry out a large-scale 2008 national community resident household survey for the first time in China. The third most important contribution of this study was that the regional differences of the key influencing factors for the community patient’s life satisfaction in China before 2009 were found for the first time. The fourth most important contribution of this study was that the robustness and universality of the major findings on the situation of China’s community health delivery system before 2009 in this study were much better than those in previous studies.

### Implications for practice

The growing community health delivery systems in most countries faced the challenge to meet the growing need for primary care in all communities, especially in medically underserved communities. Since the expectation of the whole society forced CHC to provide community medical service for the public more efficiently, more economically, more effectively, and more equally in China, the challenge for China’s community health delivery system was much more severe than the challenges for most other countries’ community health delivery systems, and then the holistic and systematic approaches to promote the community patient’s life satisfaction were more urgently needed in China than in most other countries.

In China’s future community health delivery system reform, on the basis of the major findings on the situation of China’s community health delivery system before 2009, the following inspirations on the effective approaches to promote the community patient’s life satisfaction in whole China/in various regions of China were found. The reduction of medical cost (particularly in regions with higher per capita GDP and more abundant community medical resources), the improvement of doctor-patient communication (particularly in regions with higher per capita GDP and more abundant community medical resources), the promotion of medical facility and hospital environment (particularly in regions with higher per capita GDP and more abundant community medical resources), and the improvement of medical treatment process (particularly in regions with lower per capita GDP and less abundant community medical resources) could help promote the community patient’s life satisfaction. The promotion of trust in doctor (particularly in regions with lower per capita GDP and less abundant community medical resources), the promotion of trust in prescription (particularly in regions with lower per capita GDP and less abundant community medical resources), and the promotion of trust in recommended medical examination (particularly in regions with lower per capita GDP and less abundant community medical resources) could be beneficial to the promotion of the community patient’s life satisfaction.

### Limitations of this study

Several limitations of this study should be noted. First, the response rate of the large-scale 2008 national community resident household survey was 91.83%, and then the slight deviation from the stratified sampling design might have the slight influence on the major findings of this study. Second, there might be other potential influencing factors for the community patient’s life satisfaction, although these potential influencing factors were not contained or controlled in this study, they might affect the influences of the community patient’s assessments of various major aspects of community medical service/various major aspects of the community patient’s trust in community health delivery system on life satisfaction. Third, in fact the community patient’s assessments of various major aspects of community medical service/various major aspects of the community patient’s trust in community health delivery system interrelated with each other, and this situation might affect the relative influences of the community patient’s assessments of various major aspects of community medical service/various major aspects of the community patient’s trust in community health delivery system on life satisfaction. Fourth, this study didn’t include the impact of the community health delivery system reform after 2008.

### Target readers

The findings in this study could prove useful for both practitioners in CHC and policy makers in health administration departments. Practitioners in different regions of China could find the different effective approaches to promote the community patient’s life satisfaction (through promoting the corresponding key influencing factors among major aspects of community medical service/major aspects of the community patient’s trust in community health delivery system). Policy makers in different regions of China could implement the different effective interventions for the promotion of the community patient’s life satisfaction. For example, in order to effectively promote the community patient’s life satisfaction, the improvement of medical treatment process through targeted laws, targeted policies, targeted regulations, and targeted measures should be emphasized particularly in regions with lower per capita GDP and less abundant community medical resources, while the reduction of medical cost, the improvement of doctor-patient communication, and the promotion of medical facility and hospital environment through targeted laws, targeted policies, targeted regulations, and targeted measures should be emphasized particularly in regions with higher per capita GDP and more abundant community medical resources.

## Conclusion

The reduction of medical cost (particularly in regions with higher per capita GDP and more abundant community medical resources), the improvement of doctor-patient communication (particularly in regions with higher per capita GDP and more abundant community medical resources), the promotion of medical facility and hospital environment (particularly in regions with higher per capita GDP and more abundant community medical resources), the improvement of medical treatment process (particularly in regions with lower per capita GDP and less abundant community medical resources), the promotion of trust in doctor (particularly in regions with lower per capita GDP and less abundant community medical resources), the promotion of trust in prescription (particularly in regions with lower per capita GDP and less abundant community medical resources), and the promotion of trust in recommended medical examination (particularly in regions with lower per capita GDP and less abundant community medical resources) could significantly contribute to the promotion of the community patient’s life satisfaction.

## Competing interests

The author declares that he has no competing interests.
